# Impact of silica nanoparticles incorporation on the properties of resin infiltration: an in vitro study

**DOI:** 10.1186/s12903-024-05107-7

**Published:** 2024-12-09

**Authors:** Abeer ElSayed ElEmbaby, Adham Essam Nassar, Mohamed Elshirbeny Elawsya

**Affiliations:** 1 Department of Conservative Dentistry, Faculty of Dentistry, Mansoura University, Algomhoria Street, P.O. Box 35516, Mansoura, Aldakhlia, Egypt; 2 College of Liberal Arts and Sciences, University of Florida, FL Gainesville, USA

**Keywords:** Caries infiltration, Enamel demineralization, Nano-silica, Nanoparticles, Resin infiltrant

## Abstract

**Background:**

This study evaluated the effect of nano-silica (NS) incorporation with resin infiltrant on water sorption and solubility of resin infiltrant, mineral density of demineralized enamel, and resin tags penetration.

**Methods:**

NS (Sigma-Aldrich, St Louis, Missouri, USA) was added into the resin infiltrant (ICON, DMG, Hamburg, Germany) at two concentrations by weight. The tested groups were: ICON (control), ICON + 0.2, and ICON + 0.5 (*n* = 10 per group). Water sorption and solubility were assessed using mass variation after 60 days water storage. Mineral density and surface topography were assessed using micro-Computed Tomography scans. Resin tags penetration was measured using a scanning electron microscope. Data were analyzed using one-way Analysis of Variance and Tukey’s post-hoc tests (*P* < .05).

**Results:**

ICON revealed the highest water sorption, solubility mean values (28.90, 7.61) followed by ICON + 0.2 (14.80, 4.82) and ICON + 0.5 (12.32, 0.81) respectively, and vice versa for resin tags penetration. Mineral density of demineralized enamel significantly increased after treatment with ICON + 0.2 and ICON + 0.5.

**Conclusion:**

Incorporation of NS to resin infiltrant decreased its water sorption and solubility along with enhancing the mineral density of the demineralized enamel and the penetration of resin tags.

## Introduction

Dental caries is one of the most frequent diseases that affect individuals. The aim of modern dentistry is to prevent dental caries and to remineralize enamel subsurface lesions prior to the need of restorative interventions [1]. The earliest indication of dental caries, which compromises the health and esthetics of teeth, is enamel demineralization. These lesions have the potential to be remineralized and reversed into healthy enamel tissue. The application of fluoride is usually utilized to treat these lesions. However, long-term low-level fluoride exposure can cause a number of problems with health (gastrointestinal tract problems, birth defects, bone health problems, cancer) other than dental fluorosis [2]. Therefore, it is still necessary to look for an alternative, an effective non-fluoride treatment that capable of complete curing of initial dental caries [3].

Resin infiltrant is considered an alternative strategy for arresting initial carious lesions [4, 5]. Using hydrophobic and low-viscosity light-cured resins that can pass through subsurface micropores, the infiltration approach blocks the diffusion pathway for cariogenic bacteria and their byproducts, halting the progression of the lesion [6–9].

Resin infiltrant has many advantages as: long-term closure of deeply and superficially porous demineralized regions, mechanical stabilization of demineralized enamel, preservation of sound enamel, stopping the progression of lesions by preventing further demineralization, reducing chance of developing secondary caries, immediate masking of white spot lesions, and high level of patient acceptance [10, 11]. As the esthetic improvement of white spot lesions is one of its main uses, color stability of resin infiltrant is considered a very important parameter [12–16]. However, the durability and color stability of low-viscosity light-cured resin infiltrants are considered controversial points, and this might be due to their low mechanical properties and high water sorption and solubility [17–22].

The incorporation of filler nanoparticles has been suggested by a number of earlier research to impede the deterioration of resin dental materials, as the addition of an inorganic filler to the polymer matrix could improve the physical and mechanical qualities of the material and enhance its durability [22–25]. Moreover, previous studies reported that incorporation of bioactive nanoparticles into resin infiltrant could enhance remineralization of demeniralized enamel [26–28]. A previous study [27] evaluated the properties of resin infiltrant doped with bioactive nanofibers and concluded that this experimental resin infiltrant could inhibit the demineralization of enamel and increase its hardness during a pH-cycling challenge. Another study [28] investigated the effect of nano-hydroxyapatite incorporation into resin infiltrant on remineralization of demineralized enamel and concluded that resin infiltrant incorporated with nano-hydroxyapatite effectively enhanced the artificial enamel caries in terms of mineral density and resin tags penetration.

In the field of dentistry, nano-silica (NS) is used as a reinforcing filler for resin composites, adhesives, and glass-ionomer cement, also it can be loaded with various agents [23, 29–35]. NS has many advantages as: biocompatibility, low toxicity, anti-bacterial effect, and most importantly it’s cost-effective [29–31, 36, 37]. A previous study [38] evaluated bond integrity of experimental resin adhesive filled with silica nanoparticles and concluded that the incorporation of silica nanoparticles into resin adhesive enhanced the bond strength with dentin and the formation of resin tags.

The new idea in the current study is to incorporate NS into the resin infiltrant material. The first null hypothesis tested was that the incorporation of NS with resin infiltrant had no significant effect on the physical properties (water sorption and water solubility) of resin infiltrant. The second null hypothesis tested was that the incorporation of NS with resin infiltrant had no significant effect on the mineral density of demineralized enamel. The third null hypothesis tested was that the incorporation of NS with resin infiltrant had no significant effect on the penetration of resin tags.

## Materials and methods

### Study design and sample size calculation

The study was submitted to and approved by the Dental Research Ethics Committee under protocol number A0202024CD (Faculty of Dentistry, Mansoura University). NS (Sigma-Aldrich, St Louis, Missouri, USA) was added into the resin infiltrant (ICON, DMG, Hamburg, Germany) at two concentrations by weight (0.2 wt.% and 0.5 wt.%), whereas ICON only resin infiltrant constituted the control group. The selection of NS concentrations was based on a pilot study in which all tested concentrations (0.2 wt.%, 0.5 wt.%, 0.7 wt.%, and 1 wt.%) have given positive effects, accordingly, the two lowest concentrations (0.2 wt.% and 0.5 wt.%) were chosen. The formulation groups for all tests were as follows: Group I: ICON without NS (control), Group II: ICON with 0.2 wt.% NS, Group III: ICON with 0.5 wt.% NS. Using G*power version (3.0.10) to calculate sample size based on effect size = 1.64, 2-tailed test, α error = 0.05 and power = 80% the total sample size was 9 per group (for water sorption and solubility), and 8 per group (for mineral density and resin tags penetration). So, ten specimens were prepared per group for all tests (*n* = 10).

## Water sorption and solubility

### Specimens’ preparation

Water sorption (Wsp) and water solubility (Wsl) of specimens were assessed using mass variation after 60 days water storage [20]. Using an electric digital scale (AG245 Metter, Switzerland), NS was added into the neat resin infiltrant at two concentrations (0.2 wt.% and 0.5 wt.%). NS powder was mixed with ICON resin by a dual asymmetric centrifugal laboratory vacuum mixer system (SpeedMixer.co.uk) to avoid voids. A transparent polyester Mylar strip (Maquira Industries, Av. Melvin Jones) was placed on a 1 mm thick glass microscope slide and a stainless-steel mold (8.66 mm diameter and 0.60 mm thick) was placed on top of it. The resin was applied into the mold, the top was covered by another Mylar strip and glass slide. These measurements produced a specimen with the same dimension ratio as defined by ISO 4049 standard [39]. When performing light curing, the surface of the glass slide was in direct touch with the tip of the light curing device. Light curing was achieved according to the manufacturer’s instructions by a light-emitting diode (LED) curing device (Bluephase G2; Ivoclar Vivadent, Liechtenstein) with 1200 mW/cm^2^ irradiance. The light curing was performed on both sides for 20 s each, with a constant distance of 1 mm (glass microscope slide thickness) between light curing device tip and resin. Irradiance of the light curing device was checked regularly by a light radiometer (Bluephase Meter II, Ivoclar Vivadent, Liechtenstein). The formulation groups were as follows (*n* = 10 per group): Group I: ICON without NS (control), Group II: ICON with 0.2 wt.% NS, Group III: ICON with 0.5 wt.% NS.

#### Water sorption and solubility assessment

After storage for 24 h in a dry, dark environment, the photo polymerized specimens were moved to a desiccator maintained at 37 ( ±) 1°C for 22 h. Then the specimens were transferred to a second desiccator at 25 ( ±) 1°C for 2 h, and then weighed using an electric digital scale. This procedure was repeated until a constant mass (m1), was reached. The dehydrated samples were submerged in deionized water at 37 ( ±) 1°C for 60 days, and measurements were collected again (m2) after this period. Then, the samples were reconditioned to obtain a constant dry mass (m3), following the cycle of m1 that mentioned above. The percentages of (Wsp) and (Wsl) were obtained according to the following equations:

$$\text{Wsp }= 100 \times ((\text{m}2 -\text{ m}1)/\text{m}1)$$

$$\text{Wsl }= 100 \times ((\text{m}1 -\text{ m}3)/\text{m}1)$$

## Mineral density

### Specimens’ preparation

Fifteen human sound permanent maxillary first premolars freshly extracted due to orthodontic treatment were collected. Any soft tissue and calculus deposits were meticulously removed by employing an ultrasonic scaler during the teeth-cleaning process. The teeth were polished with a rubber cup and non-fluoride pumice. Then the teeth were rinsed with tap water and stored in a 0.5% Chloramine T solution until they were ready for use. Every tooth underwent a central sectioning in a mesio-distal manner, parallel to its long axis with an Isomet low-speed saw (Buehler, Lake Bluff, IL, USA) into 2 halves, giving 30 specimens. Then, the roots were removed (1 mm cervical to CEJ). A thin layer of acid-resistant varnish was applied to each specimen, leaving a uniform enamel window (2 × 2 mm) located in the central portion of the lingual and buccal surfaces. Before demineralization, all samples were kept in distilled water for 24 h.

### Demineralization procedure

Subsurface caries lesions were formed using a fresh demineralizing solution that was changed daily. The demineralizing solution consisted of 2 mM Ca (Ca [NO_3_]_2_), 2 mM PO_4_ (KH_2_PO_4_), and 75 mM acetate at pH 4.3. The samples were cycled twice daily for 21 days at 37 ( ±) 1°C between the demineralizing solution and artificial saliva consisted of 1.5 mM calcium chloride, 0.9 mM monosodium phosphate, and 0.15 M potassium chloride at pH 6.8 [28, 40, 41].

### Treatment procedure

After demineralization, the thirty samples were blindly divided into three equal groups (*n* = 10 per group), according to the treatment material: Group I: ICON without NS (control), Group II: ICON with 0.2 wt.% NS, Group III: ICON with 0.5 wt.% NS. First, etchant (ICON Etch, DMG, Hamburg, Germany) was applied over the demineralized area for 2 min. This was followed by water rinsing and air drying. Then, ethanol (ICON Dry, DMG, Hamburg, Germany) was applied for 30 s and air dried. Using the provided sponge applicator, Infiltrant was rubbed on and left for 3 min then light cured for 20 s. This step was repeated with another layer of infiltrant being placed and rubbed on for additional 1 min, and again cured for 20 s. All treatment procedures were achieved according to manufacturer’s instructions. Prior to micro-Computed Tomography (micro-CT) scanning, all samples were kept in artificial saliva for 24 h.

### Scanning and image processing

A micro-CT scanner system (Skyscan 1172, Bruker Micro-CT, Kontich, Belgium) was used to scan the specimens. The scanning parameters were: voltage – 80 kV, current – 100 μA, integration time – 3.63 s, isotropic resolution – 9 μm with rotation at 360° and 1° steps. By scanning hydroxyapatite reference phantoms with known mineral densities of 0.25 and 0.75 g/cm^3^, the calibration of mineral density was obtained. A software (NRecon, Bruker Micro-CT, Belgium version 1.6.4.8, Skyscan 2011) was used to reconstruct the raw images. Mineral density was recorded using a software (CTAn, Bruker Micro-CT, Belgium version 1.11.10.0‏ 64-bit, Skyscan 2003–11), and another software (CTVol, Bruker Micro-CT, Belgium version 2.2.1.0 64-bit, Skyscan 1983–2010) was employed for viewing 3D images. For mineral density assessment, five sections (9 μm each) at three randomly selected levels (within the 2 × 2 mm window) were identified. To assess mineral density at three uniformly distant points, a uniform region of interest with a depth of approximately 100 μm was used, taking into consideration the trans-axial view. A mean mineral density value was calculated from each combined five sections at three random levels. Values for mineral density (g/cm^3^) were obtained through gray scale values [42–44]. For each sample in all groups, mineral density was obtained three times: before demineralization, after demineralization, and after the application of resin infiltrant. Using the formula below, the percent change in mineral density was determined:

Change in mineral density = [(mineral density after application of resin infiltrant—mineral density after demineralization) / mineral density after demineralization] × 100.

Moreover, micro-CT scans were captured to evaluate the surface topography of enamel in all groups at all stages: before demineralization, after demineralization, and after application of resin infiltrant.

#### Resin tags penetration measurement

After mineral density assessment, all samples were analyzed by scanning electron microscope (SEM) to measure resin tags penetration. Prior to scanning, each specimen was sectioned centrally in an occluso-gingival direction, parallel to its long axis, with an Isomet low-speed saw (Buehler, Lake Bluff, IL, USA) through the center of the treated area to expose the tooth-infiltrant interface. For all groups, one half from each specimen was included (*n* = 10 per group). The specimens were sonicated for 5 min, then they were polished sequentially with 400, 600, 800, and 1200 grit sandpapers using a polishing machine (Eco-Met™ 30 variable speed grinder polisher, Buehler, Lake Bluff, IL, USA). A final polish using aluminum oxide paste (1μm) was carried out, and then all samples were sonicated again.

**Fig. 1:**
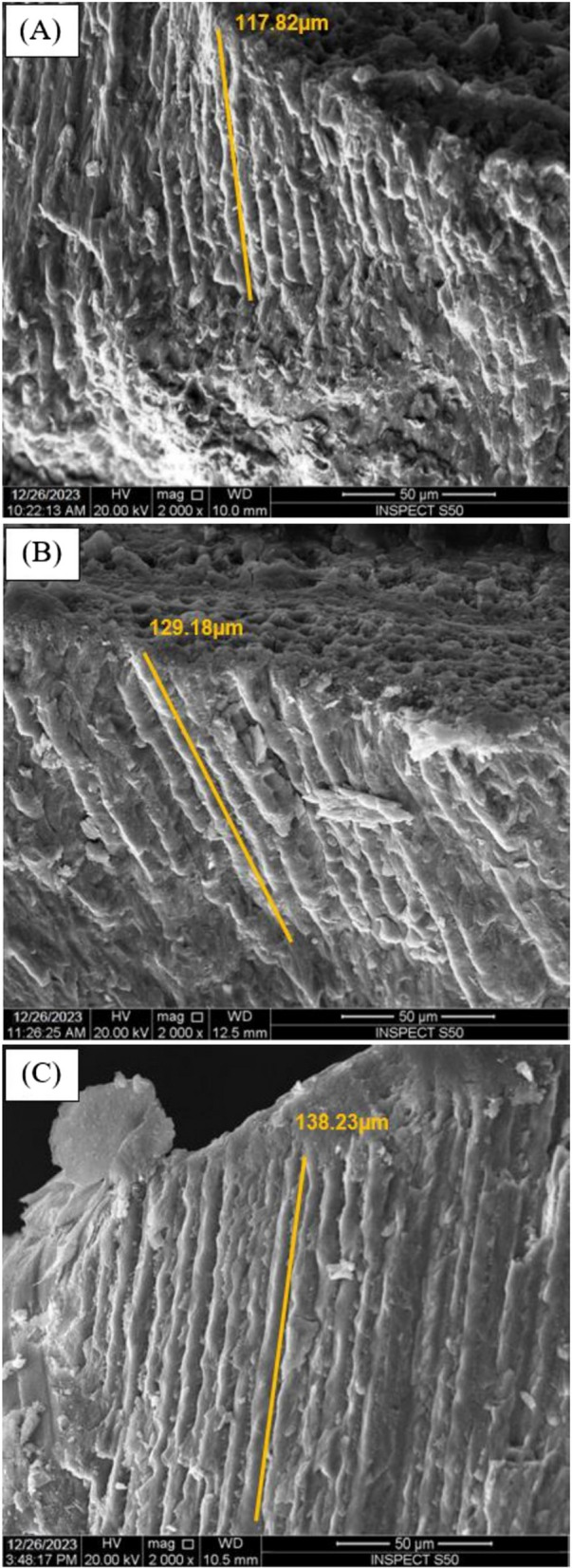
Representative SEM micrographs at × 2,000 magnification showing resin tags penetration measurements for all tested groups; **(A)** Group I: ICON without NS (control), **(B)** Group II: ICON with 0.2 wt.% NS, and **(C)** Group III: ICON with 0.5 wt.% NS

For SEM, double-faced cohesive carbon tapes were used to mount the samples on metallic stubs and the samples were then gold-coated. The SEM operated at a working distance of roughly 10 mm and an accelerating voltage of 20 kV. For resin tags penetration measurements, at least ten resin tag lengths were measured in micrometers (μm) and the average was calculated for each sample (Fig. 1).

#### Statistical analysis

Statistical analyses were performed using SPSS 22 software (IBM SPSS Inc., Chicago, IL, USA). Shapiro–Wilk test revealed that the data had a parametric distribution. Therefore, one-way analysis of variance (ANOVA) was used for analyzing the data followed by Tukey’s post-hoc test for multiple comparisons. The level of significance was set at *P* < 0.05 in all analyses.

## Results

### Water sorption results

One-way ANOVA test indicated a statistically significant difference between water sorption values of the tested groups (*P* < 0.0001). ICON group revealed the highest water sorption mean value followed by ICON + 0.2 group and ICON + 0.5 group respectively. Tukey’s post-hoc test indicated a statistically significant difference between all tested groups (*P* < 0.0001). Water sorption % mean values ± standard deviations (SDs) of all tested groups and Tukey’s post-hoc test results are presented in Table 1.

**Table 1:** Water sorption % and water solubility % means ± SDs of all tested groups with Tukey’s post-hoc test results

	**Group I** **(ICON without NS)**	**Group II** **(ICON with 0.2 wt.% NS)**	**Group III** **(ICON with 0.5 wt.% NS)**
Water sorption	28.90 ± 0.61 A	14.80 ± 0.81 B	12.32 ± 1.39 C
Water solubility	7.61 ± 0.46 A	4.82 ± 0.54 B	0.81 ± 0.07 C

–Different capital letters in the same raw indicate statistically significant differences

### Water solubility results

One-way ANOVA test indicated a statistically significant difference between water solubility values of the tested groups (*P* < 0.0001). ICON group revealed the highest water solubility mean value followed by ICON + 0.2 group and ICON + 0.5 group respectively. Tukey’s post-hoc test indicated a statistically significant difference between all tested groups (*P* < 0.0001). Water solubility % mean values ± SDs of all tested groups and Tukey’s post-hoc test results are presented in Table 1.

### Mineral density results

**Fig. 2:**
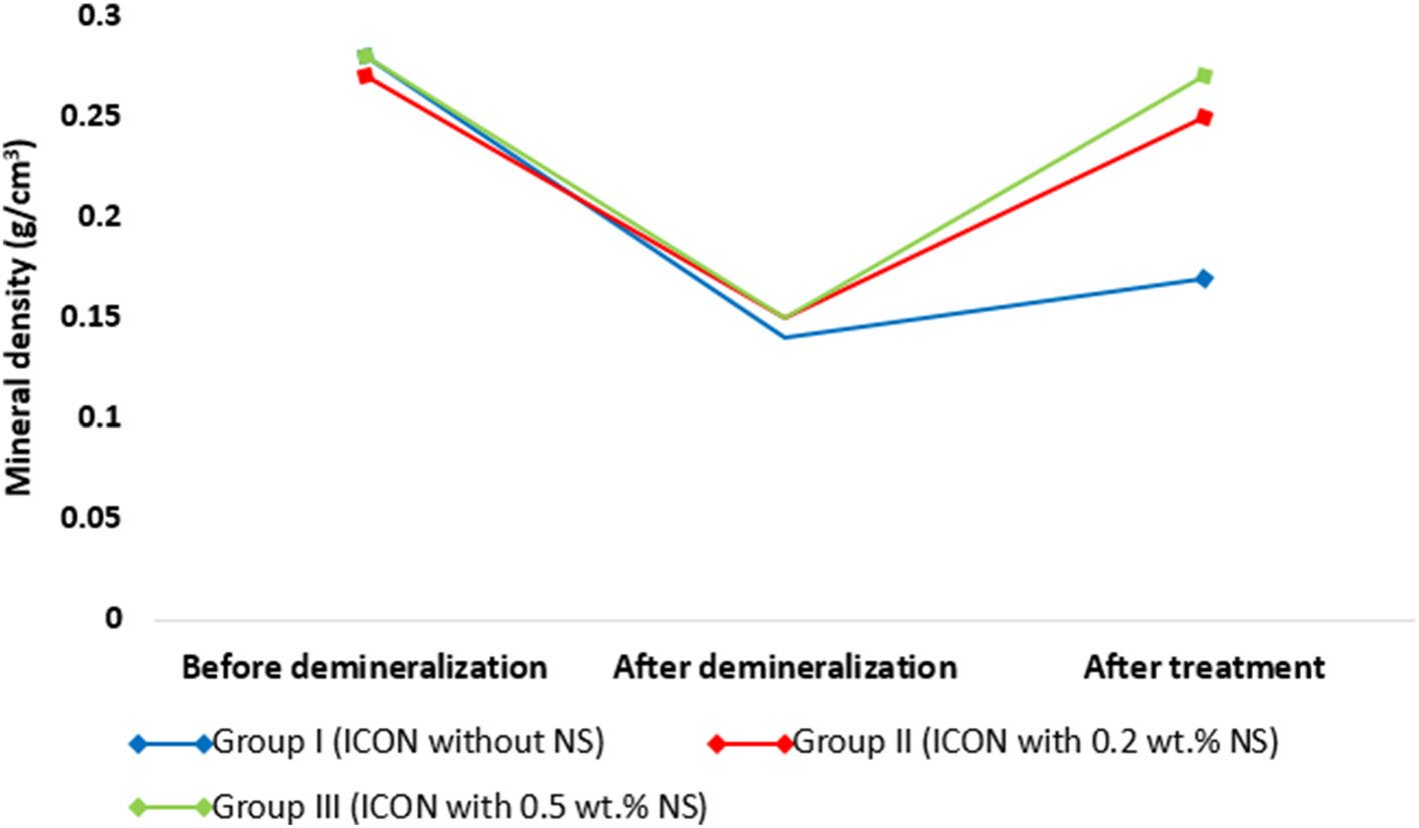
A line chart represents the changes in mineral density in all tested groups at the three stages: before demineralization, after caries induction, and after treatment

Mineral density mean values ± SDs of all tested groups at the three stages (before demineralization, after caries induction, and after treatment) are presented in Table 2. Also, the percent change in mineral density mean values ± SDs of all tested groups and Tukey’s post-hoc test results are presented in Table 2. Changes in mineral density in all tested groups at the three stages (before demineralization, after caries induction, and after treatment) are presented in Fig. 2.

**Table 2:** Mean values ± SDs of mineral density (g/cm^3^) at all stages and percent change in mineral density for all tested groups with Tukey’s post-hoc test results

Stage	Group I (ICON without NS)	Group II (ICON with 0.2 wt.% NS)	Group III (ICON with 0.5 wt.% NS)
Before demineralization	0.28 ± 0.01 ^A^	0.27 ± 0.01 ^A^	0.28 ± 0.01 ^A^
After demineralization	0.14 ± 0.02 ^B^	0.15 ± 0.01 ^B^	0.15 ± 0.02 ^B^
After treatment	0.17 ± 0.01 ^Bb^	0.25 ± 0.04 ^Aa^	0.27 ± 0.02 ^Aa^
*P* value	< .0001	< .0001	< .0001
Percent change %	23.90 ± 12.64 ^b^	75.08 ± 31.18 ^a^	90.33 ± 37.07 ^a^

^A,B^Different capital letters in same column indicate statistically significant differences

^a,b^Different lowercase letters in same row indicate statistically significant differences

Regarding ICON group, there was a significant drop from baseline (before demineralization) after demineralization (*P* < 0.0001), and there was no statistically significant difference between after demineralization and after treatment (*P* = 0.092).

Regarding ICON + 0.2 group, there was a significant decrease from baseline after demineralization (*P* < 0.0001), and a significant increase from demineralization after treatment (*P* < 0.0001). There was no statistically significant difference between baseline and after treatment (*P* = 0.187).

Regarding ICON + 0.5 group, there was a significant decrease from baseline after demineralization (*P* < 0.0001), and a significant increase from demineralization after treatment (*P* < 0.0001). There was no statistically significant difference between baseline and after treatment (*P* = 0.870).

After treatment in all groups, there was no statistically significant difference between ICON + 0.2 group and ICON + 0.5 group (*P* = 0.291), but there was a statistically significant difference between them and ICON group (*P* < 0.0001).

Regarding the percent change in mineral density in each group, there was no statistically significant difference between ICON + 0.2 group and ICON + 0.5 group (*P* = 0.475), but there were statistically significant differences between ICON group and ICON + 0.2 group (*P* = 0.001), and between ICON group and ICON + 0.5 group (*P* < 0.0001).

### Enamel surface topography

**Fig. 3:**
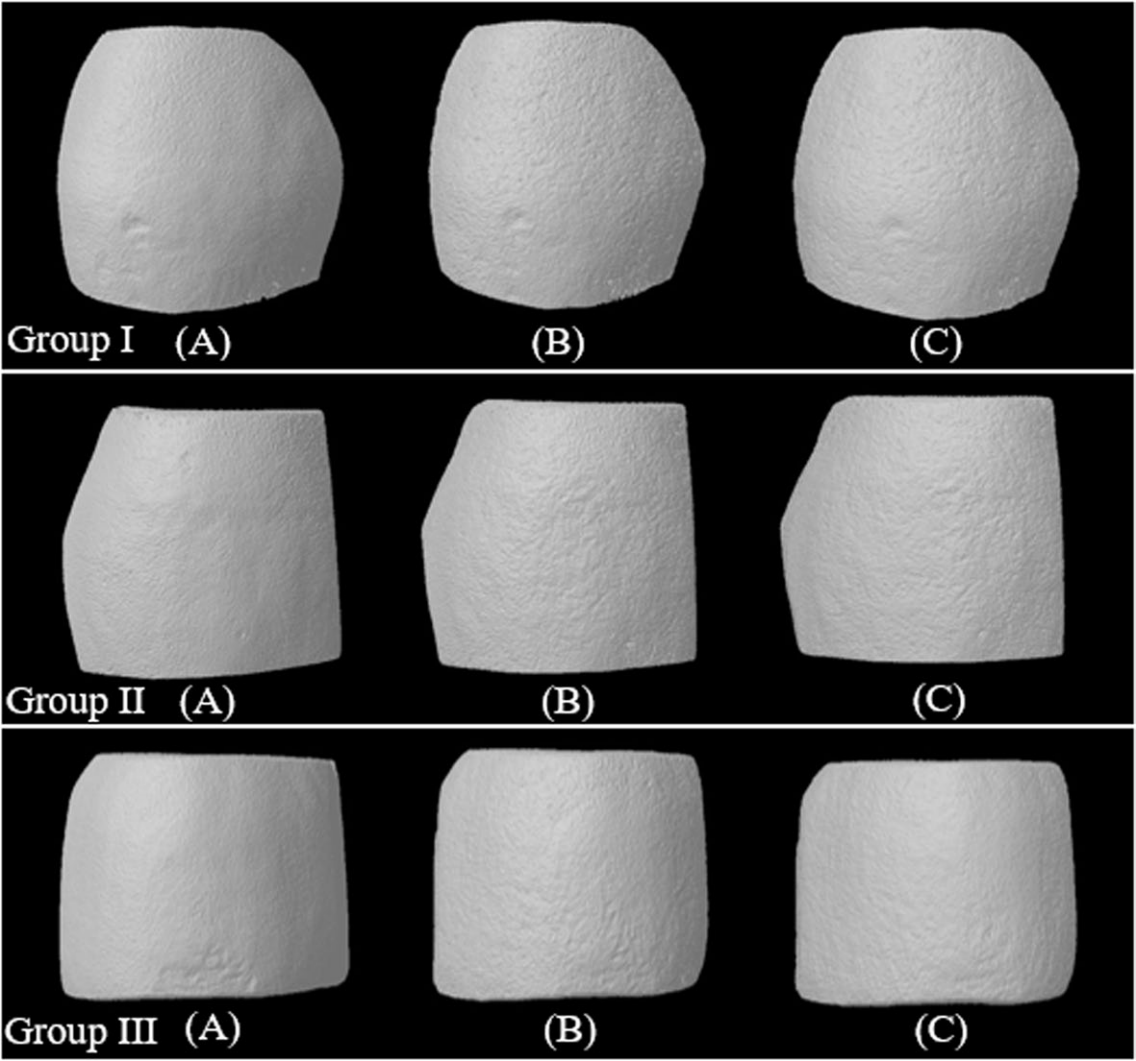
Micro-CT scans of representative enamel surfaces topographies at the three stages: (A) before demineralization, (B) after caries induction, and (C) after treatment, of all tested groups; Group I: ICON without NS (control), Group II: ICON with 0.2 wt.% NS, and Group III: ICON with 0.5 wt.% NS

Changes in enamel surface topography were clear at the three stages as shown by micro-CT scans (Fig. 3). Before demineralization, normal and smooth enamel surface was shown in all groups. After demineralization, rough and irregular enamel surfaces were evident. After the treatment, enamel surfaces of all groups were more regular than the demineralized enamel surfaces but less regular than normal enamel surfaces. Treated enamel surfaces with ICON + 0.5 were more regular than treated enamel surfaces with ICON and ICON + 0.2.

### Resin tags penetration results

One-way ANOVA test indicated a statistically significant difference between resin tags measurements values of the tested groups (*P* < 0.0001). ICON + 0.5 group revealed the highest resin tags measurements mean value followed by ICON + 0.2 group and ICON group respectively. Tukey’s post-hoc test indicated a statistically significant difference between all tested groups (*P* < 0.0001). Resin tags measurements mean values ± SDs of all tested groups and Tukey’s post-hoc test results are presented in Table 3.

**Table 3:** Resin tags measurements mean values (μm) ± SDs of all tested groups with Tukey’s post-hoc test results

Group I (ICON without NS)	Group II (ICON with 0.2 wt.% NS)	Group III (ICON with 0.5 wt.% NS)
116.90 ± 4.23 C	128.80 ± 3.85 B	138.10 ± 4.75 A

–Different capital letters indicate statistically significant differences

## Discussion

The current study's findings may have implications for clinical practice. Since it has been studied on ICON discs, it has been speculated that although ICON can resolve the first esthetic issue related to demineralization, the resin may eventually become more unstable. In the current study, incorporation of NS with resin infiltrant improved the physical properties (water sorption and water solubility) of resin infiltrant, mineral density of demineralized enamel, and penetration of resin tags, so all null hypotheses were rejected.

Several researchers have investigated the use of nanoparticles in restorative and preventive dentistry since the advent of nanotechnology [23, 27, 28, 30, 33, 34]. In order to investigate its effect on the mechanical characteristics, bond strength, and remineralization potential, researchers have previously incorporated various filler nanoparticles, such as hydroxyapatite, silica, calcium-rich zeolite, and calcium phosphate into dental adhesives, resin composites, and resin infiltrants [24, 26, 28, 38]. These investigations have demonstrated that the mechanical characteristics, remineralization potential, bond strength, and durability of these materials have all been improved by adding these fillers.

Recently, there has been an increase in the usage of NS in dentistry and it is used as fillers in adhesives, resin composites, and glass-ionomer cement. NS has many advantages as: biocompatibility, low toxicity, and most importantly it’s cost-effective [23, 29–31, 33–35, 38]. In order to obtain its benefits with resin infiltrant, we decided to investigate the impact of NS incorporation on the properties of resin infiltrant. According to previous studies [36, 37], adding NS to the resin matrix at concentrations higher than 1 wt.% could increase the agglomeration tendency of nanoparticles and reduce homogeneity within the matrix. In addition, there are many studies found that the properties of dental adhesives could be enhanced by incorporating silica nanoparticles at low concentrations as opposed to higher concentrations. This is because lower concentrations of nanoparticles allow for the formation of a resilient structure through homogeneous dispersion, whereas higher concentrations tend to form clusters that adversely affect adhesive properties [32, 38, 45–48]. Therefore, the two lowest concentrations (0.2 wt.% and 0.5 wt.%) whose effectiveness was tested in a prior pilot study, were chosen for the current study.

Stability against disintegration and dissolution is one of the most crucial characteristics affecting the longevity of dental materials in the oral cavity. Undesirable structural alterations and functional disruptions may arise from water sorption and solubility phenomena of dental resin materials. The physical–chemical properties of all components of resin composite materials, such as the resulting polymer network's three-dimensional structure, the free volume trapped inside the structure, and the network's hydrophilic nature and solubility parameter, all have a significant impact on the water sorption and solubility of these materials [20].

The most prevalent chemical components of resin infiltrant materials are the resin monomers. The ester group of both acrylates and methacrylates monomers is susceptible to water breakdown (hydrolysis). Due to its hydrophilicity, it could absorb water, increasing the susceptibility of the cured resin's monomers to hydrolysis [21].

Although tetraethylene glycol dimethacrylate (TEGDMA) demonstrated the best ability to infiltrate deeply into the non-cavitated carious lesions, TEGDMA has the highest water sorption capability, followed by bisphenol A diglycidyl methacrylate (BisGMA) and by urethane dimethacrylate (UDMA) [21]. Therefore, the primary component of the resin infiltrant used in the present study is TEGDMA resin monomer. This might be the cause of the ICON's propensity for water sorption and solubility. Furthermore, the lack of filler in the resin infiltrant ICON makes it more susceptible to water sorption and solubility than the groups containing NS. As inorganic fillers reduce the proportion of a polymeric matrix in a resin-based dental material's composition, resulting in less shrinkage and hydrolytic degradation. In the current study, when NS (0.2 wt.% or 0.5 wt.%) was added to ICON, the tested resin's water sorption and solubility percentages drastically decreased. This might have happened because of the presence of NS, which could have decreased the resin's hydrophilicity. A previous study conducted by Sfalcin et al. [22] has similar findings.

Since the pH cycling model is more reliable for clinical situation and is regarded as an appropriate method of stimulating the caries process, it was employed in the current study to achieve so [28, 41]. There are several approaches used to assess tooth mineral density, in both the direct and indirect manners. Micro-CT is a nondestructive method that can evaluate the concentration of minerals in three dimensions at the micron level. This method can distinguish between sound, demineralized, and remineralized enamel for enamel body and surface with high accuracy [42–44]. The samples are not destroyed, and they can be rescanned in order to compare and assess the sample's mineral density at all stages of the experiment. Consequently, this approach can determine the gain or loss of mineral content with great accuracy. In the current study, the incorporation of NS into ICON (ICON + 0.2 and ICON + 0.5) increased the mineral density of the treated specimens significantly in comparison with the demineralized specimens. A previous study conducted by Besinis et al. [33] has similar findings.

In the current study, the incorporation of NS to ICON (ICON + 0.2 and ICON + 0.5) improved resin tags penetration in comparison with ICON only. These results are consistent with previous study results that added the same commercial NS product used in the current study to a dental adhesive and attributed these results to the spherical shape of NS particles [38]. As spherical shape of filler particles may have a lubricating effect on the material, facilitating easy flow with minimal viscosity alteration. Conversely, amorphous particles—particles lacking a distinct shape—might obstruct the flow and raise the viscosity [25].

In the current study, although incorporation of NS with resin infiltrant has increased the mineral density of demineralized enamel, its remineralization potential should be investigated in further studies by elemental analysis at infiltrant/enamel interface. Owing to the restricted focus of this study, additional comprehensive researches are required to examine the potential chemical reaction between the NS molecule and the resin infiltrant components in order to comprehend any potential protective effects of NS. In addition, only two concentrations of NS (0.2 wt.% and 0.5 wt.%) have been evaluated in this study with resin infiltrant, so further studies with different concentrations are needed. Also other properties of resin infiltrant incorporated with NS such as bacterial adhesion, color stability, surface roughness, gloss, and microhardness, should be investigated.

## Conclusions

Within the limitations of this current study, it was concluded that:

1. Incorporation of NS with ICON enhanced its stability and decreased its water sorption and solubility, in addition to enhancing penetration of resin tags.
2. Incorporation of NS with ICON increased the mineral density of demineralized enamel to be healed rather than obturating and sealing the porosity of the demineralized tissue.
3. Although both concentrations of NS (0.2 wt.% and 0.5 wt.%) enhanced all tested properties, the higher concentration (0.5 wt.%) was better.

## Data Availability

The data that support the findings of this study are available from the corresponding author upon reasonable request.
